# Defending the genome from the enemy within: mechanisms of retrotransposon suppression in the mouse germline

**DOI:** 10.1007/s00018-013-1468-0

**Published:** 2013-09-18

**Authors:** James H. Crichton, Donncha S. Dunican, Marie MacLennan, Richard R. Meehan, Ian R. Adams

**Affiliations:** MRC Human Genetics Unit, MRC Institute of Genetics and Molecular Medicine, University of Edinburgh, Western General Hospital, Crewe Road, Edinburgh, EH4 2XU UK

**Keywords:** Retrotransposon, Germ cell, Genome defence, Epigenetics, DNA methylation, Mouse, Meiosis

## Abstract

The viability of any species requires that the genome is kept stable as it is transmitted from generation to generation by the germ cells. One of the challenges to transgenerational genome stability is the potential mutagenic activity of transposable genetic elements, particularly retrotransposons. There are many different types of retrotransposon in mammalian genomes, and these target different points in germline development to amplify and integrate into new genomic locations. Germ cells, and their pluripotent developmental precursors, have evolved a variety of genome defence mechanisms that suppress retrotransposon activity and maintain genome stability across the generations. Here, we review recent advances in understanding how retrotransposon activity is suppressed in the mammalian germline, how genes involved in germline genome defence mechanisms are regulated, and the consequences of mutating these genome defence genes for the developing germline.

## Introduction

Maintaining genetic stability through the generations is key for the survival of all species. If the germline mutation rate is too low, there will not be sufficient variation within the species to adapt and survive over evolutionary time; yet, if the germline mutation rate is too high, the accumulation of deleterious mutations will impact on viability. One of the major drivers of genetic change in the genome is retrotransposons. Retrotransposons are highly abundant mobile genetic elements that account for around 40 % of the sequenced mammalian genome [1, 2]. Retrotransposons contribute to genome instability by acting as sites for recombination-mediated chromosomal deletions and re-arrangements by influencing expression of nearby genes, and by causing mutations when new retrotransposition events disrupt pre-existing genetic information in the host genome [3].

For retrotransposons to be successful, retrotransposition must occur in the germ cells, or in the pluripotent cells from which germ cells arise. Germ cells and pluripotent cells have evolved multiple genome defence mechanisms to limit the mutagenic activity of these mobile genetic elements [4–6]. A number of genes involved in suppressing retrotransposon activity in the mammalian germline have been identified, and the ways that these genome defence mechanisms are regulated and interact to provide an effective defence against retrotransposons are starting to be understood. In this review, we will describe the different types of retrotransposons in mammalian genomes, the mechanisms that germ cells and pluripotent cells use to suppress the activity of these elements, and the phenotypic defects that arise in the developing germ cells when germline genome defence genes are mutated.

## Mammalian retrotransposons

**Fig. 1:**
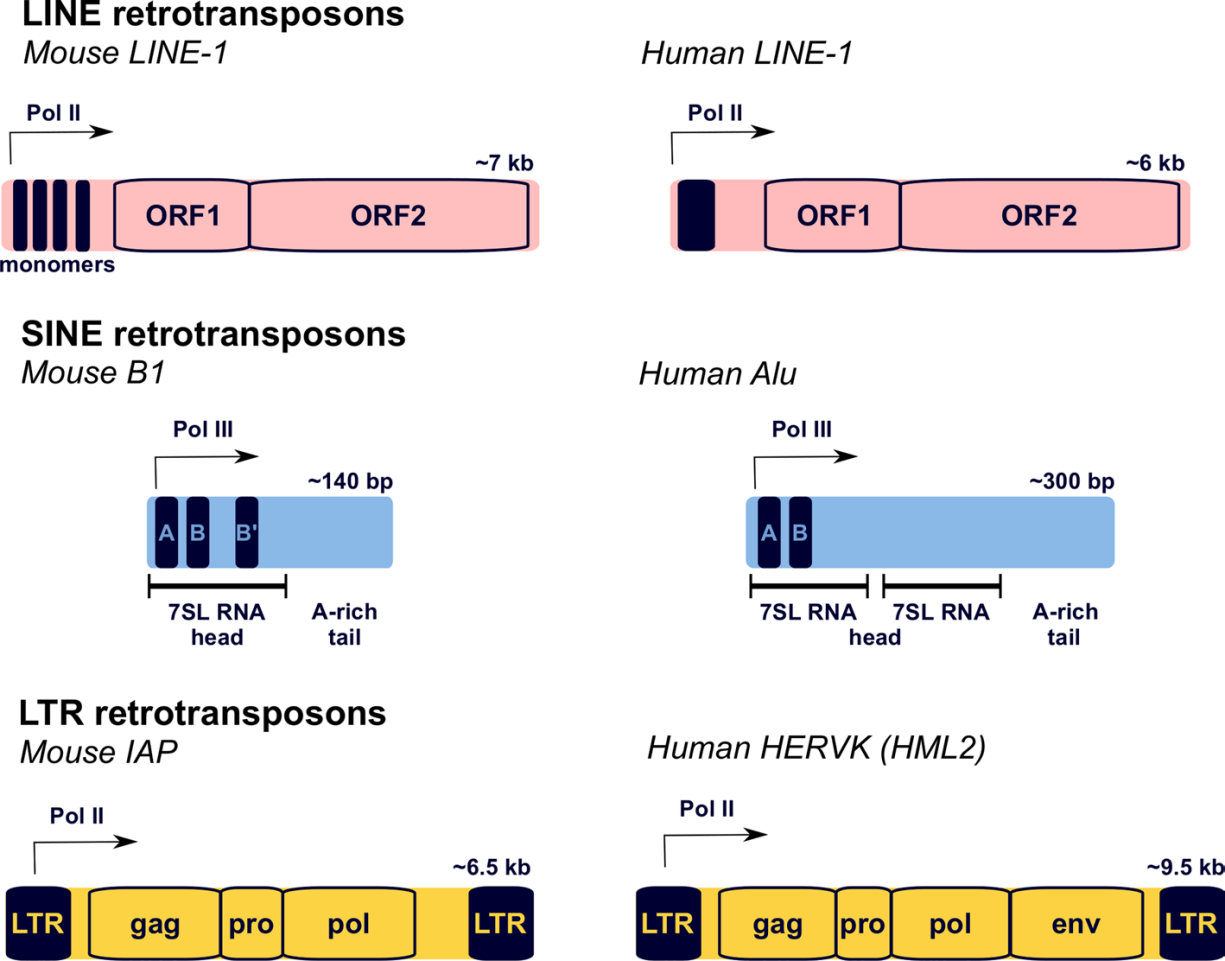
Structure of the major mammalian retrotransposons. Mouse and human examples of the three different classes of mammalian retrotransposon (*LINE*, *SINE*, *LTR*) are shown. Transcription regulatory regions are indicated with *filled rectangles*, and the main protein coding regions with *open rectangles*. Transcriptional start sites are shown with an *arrow*. Some LTR retrotransposons, e.g. IAP, have lost the *env* gene present in their infectious progenitors [241]. *LTR* long terminal repeat

The mammalian genome contains three major classes of retrotransposon: long interspersed nuclear element (LINE), short interspersed nuclear element (SINE), and long terminal repeat (LTR) retrotransposons (Fig. 1) [1, 2]. Each of these retrotransposon classes has different characteristics and properties.

### LINE retrotransposons

LINE-1, which accounts for ~17 % of the sequenced genome, is the major family of LINE retrotransposons in mammals. Most copies of LINE-1 in mammalian genomes are 5′-truncated, probably due to incomplete reverse-transcription of this element during retrotransposition [1, 2]. Full-length mouse and human LINE-1 elements are around 6–7 kb in length and contain a variable internal promoter driving expression of these elements (Fig. 1). The internal promoter differs significantly in sequence between mouse and human LINE-1 elements, and between different LINE-1 subfamilies within species [7–9]. LINE-1 promoters may have been co-opted from host sequences, and differences between LINE-1 promoter sequences could lead to variations in the transcription factor requirements and regulation of these elements. The transcribed LINE-1 mRNA encodes two proteins, ORF1 and ORF2, that are each required for LINE-1 retrotransposition [10, 11]. ORF1 encodes a ~40-kDa nucleic acid-binding protein containing a central RNA recognition motif that has nucleic acid chaperone activity, while ORF2 encodes a ~150-kDa endonuclease and reverse-transcriptase [12–15]. Both ORF1 and ORF2 are thought to preferentially interact with the same mRNA molecule from which they are translated, which generates a strong *cis*-preference for that encoding mRNA to be retrotransposed [16]. LINE-1 elements are still retrotranspositionally active in mammals, and spontaneous de novo integrations of LINE-1 elements have been reported to cause phenotypic changes and disease in both mice and humans [17–19].

### SINE retrotransposons

Short interspersed nuclear element elements are non-autonomous retrotransposons that make up around 10 % of the sequenced mammalian genome [1, 2]. SINE elements are derived from a broad range of small cellular RNA polymerase III transcripts including 7SL RNA, 5S rRNA, and tRNAs, and rely on LINE-1-encoded proteins, particularly LINE-1 ORF2, to catalyse their retrotransposition [20–22]. SINE elements typically contain an internal RNA polymerase III promoter and are therefore regulated quite differently from the LINE-1-encoded proteins that allow them to retrotranspose. The most prominent families of SINE elements in human and mouse genomes are the Alu and B1 elements, respectively. Human Alu (~300 bp) and mouse B1 (~140 bp) elements are derived from the signal recognition particle 7SL cellular RNA, but subsequent re-arrangements and duplications has led to these elements having quite different structures (Fig. 1) [23]. SINE elements can also be incorporated into cellular transcripts that run through their integration sites, which can provide a route for new variants of these elements to arise [24]. De novo insertions of SINE elements have been identified as potentially causative mutations in both human and mouse genetic disease [17, 25]. Even though SINE elements require LINE-1-encoded proteins for their mobilisation, the germline SINE retrotransposition rate in humans is estimated to be around five times higher than that of LINE-1 [17].

### LTR retrotransposons

Long terminal repeat retrotransposons, also known as endogenous retroviruses (ERVs), have a typical retroviral structure with protein-coding gag, pol, pro and sometimes env genes flanked by long terminal repeats that act as promoters (Fig. 1) [26]. There are around 150 different types of ERV in a typical mammalian genome, classified into ERV1, ERVK, ERVL and MaLR families depending on their phylogenetic relationship. Like LINE-1 elements, LTR retrotransposons require the activity of the proteins that they encode in order to retrotranspose [27]. However, some LTR retrotransposons are non-autonomous and rely on proteins encoded by a different element to catalyse their retrotransposition [28]. LTR retrotransposons make up around 9 % of the human and mouse genomes; however, most of these LTR retrotransposons are lineage-specific elements that have arisen after the mouse and human lineages diverged from a common ancestor 65–75 million years ago [2]. Different types of LTR retrotransposon have been successful in colonising mouse and human genomes; ERVK elements are ten times more abundant in mouse than in human genomes, whereas human ERV1 elements are four times more abundant than their mouse counterparts [2]. With the possible exception of HERVK (HML2) elements, the LTR retrotransposons that currently exist in the human genome do not appear to be retrotranspositionally active [26], and de novo insertions of LTR retrotransposons have not yet been identified as mutant alleles in human genetic disease [18]. In contrast, LTR retrotransposons are retrotranspositionally active in the mouse genome, and de novo insertions of ERV1, ERVK, ERVL and MaLR LTR retrotransposons have all been associated with spontaneous mutations in mice [18].

## Retrotransposon expression in germ cells and pluripotent cells

**Fig. 2:**
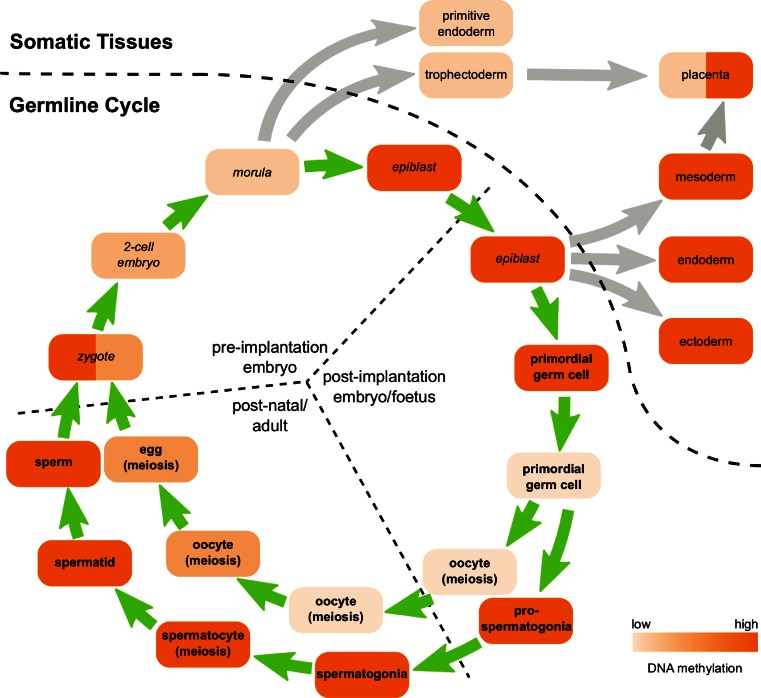
Overview of the mouse germline cycle. Schematic overview of germ cell development in mice. Pluripotent cells are indicated in *italicised* text and germ cells in *bold* text. DNA is passed between germ cells and pluripotent cells through the generations in a germline cycle (*green arrows*). Differentiation into somatic tissues is indicated by *grey arrows*. The level of DNA methylation at different stages of development is indicated by the level of *shading*

For retrotransposons to be successful, they must be expressed and functional in developing germ cells, or in pluripotent cells, during the germline cycle (Fig. 2). De novo retrotransposition events in the germline cycle have been proposed to shape the genomic landscape through mediating chromosomal re-arrangements and by acting as a reservoir for the the emergence of new genes, in addition to providing a source of genetic variation within and between individuals [29, 30]. As the chromatin environment, transcription factor availability and post-transcriptional regulation of gene expression all vary significantly during the germline cycle, different retrotransposons have evolved to target different stages of germ cell development. The resulting variety of complex and dynamic retrotransposon expression patterns means that exaptation of retrotransposon sequences can play a role in the evolution of gene expression networks at multiple points within the germline cycle [29, 31–33].

### Retrotransposon expression in pre-implantation embryos

During pre-implantation development, the zygote undergoes a series of mitotic divisions to generate a ball of pluripotent cells known as a morula (Fig. 2). The morula compacts and cavitates to generate a blastocyst possessing trophectoderm, primitive endoderm and epiblast layers. The trophectoderm and primitive endoderm layers will give rise only to extra-embryonic structures, whereas the epiblast cells retain pluripotency and will give rise to all the tissues in the embryo, including the germ cells, after implantation (Fig. 2) [34]. Pre-implantation development only takes around 4 days in mice, but shows dynamic changes in retrotransposon expression. For example, RNA transcripts belonging to one of the most abundant LTR retrotransposons in the mouse genome, IAP elements, are present at high levels in fully-grown oocytes, decrease in 1-cell embryos, then increase again during development to the blastocyst stage [32, 35, 36]. These IAP element transcripts are competent to generate A-type retroviral-like particles, whose abundance follows similar dynamics during these stages of development [36]. In contrast, epsilon-type retroviral-like particles, which are encoded by MuERVL ERVL LTR retrotransposons, are not present in fully-grown oocytes, transiently increase in abundance in 2-cell embryos, then disappear as pre-implantation development proceeds [36]. Again, the changes in epsilon-type retroviral-like particle abundance are mirrored by changes in abundance of MuERVL transcripts that encode these elements [32, 37]. The MT MaLR LTR retrotransposon transcripts exhibits yet another distinct expression pattern during pre-implantation development and are highly abundant in mouse oocytes but sharply decrease in abundance as pre-implantation development proceeds [32]. Thus, different types of retrotransposon have evolved to take advantage of the dynamic chromatin modifications and transcription factor profiles present at these stages of development. Interestingly, chimaeric transcripts originating from retrotransposon promoters but spliced onto host genes are present in oocytes and pre-implantation embryos [32], suggesting that mammalian hosts are co-opting retrotransposons to drive gene expression during these stages of development.

### IAP LTR retrotransposon expression during germ cell development

Germ cell development starts after implantation when the pluripotent epiblast differentiates into primordial germ cells in addition to the endoderm, mesoderm and ectoderm somatic tissues during gastrulation at E6.5–E7.5 (Fig. 2). The primordial germ cells proliferate and migrate to the genital ridges, colonising these structures around E10.5, and differentiate into meiotic oocytes or quiescent prospermatogonia by E13.5–E14.5 (Fig. 2). In males, the quiescent prospermatogonia can differentiate into spermatogonial stem cells a few days after birth, which will give rise to cells progressing through spermatogenesis (proliferating spermatogonia → meiotic spermatocytes → post-meiotic spermatids → sperm) throughout the adult life of the animal (Fig. 2). In females, the oocytes that initiate meiosis in the foetus undergo meiotic arrest a few days after birth, and groups of these arrested oocytes are selected to grow and mature during each oestrus cycle. Oocyte meiosis is not completed until the ovulated egg is fertilised to generate a zygote (Fig. 2) [4]. The advent of next generation sequencing technologies, and their application to analyse the transcriptome of small numbers of cells, is likely to generate a wealth of data about genome-wide retrotransposon transcript levels at different stages of the germline cycle [38, 39]. However, many stages of germ cell development have not yet been extensively analysed, and much of our understanding of retrotransposon expression during gametogenesis comes from studies on specific elements.

One of the best-studied LTR retrotransposons in mice is the IAP element. The IAP LTR drives transcription of a *LacZ* reporter preferentially in germ cells rather than somatic cells [40], and contains binding sites for a number of widely-expressed transcription factors, including YY1, SP1, CREB1 and glucocorticoid receptors [41]. Thus, the germline-specific expression of IAP elements does not appear to be caused by germ cell-specific transcription factors. Rather, the preferential expression of IAP elements in the germline appears to be a consequence of IAP element DNA being hypomethylated in germ cells, but methylated and silenced in somatic cells [42–45]. Within the germline, IAP LTR-driven expression of a *LacZ* reporter is restricted to a fairly small window of germ cell development, with activity detectable in quiescent male foetal germ cells from E16, and in undifferentiated spermatogonia in adult testes [40]. IAP DNA is hypomethylated at these stages [39, 40, 46–48]. Although the IAP LTR is sufficient to drive germline expression of a *LacZ* reporter, it does not recapitulate endogenous IAP transcription in adult liver [49] nor in pre-implantation embryos [32, 35, 36]. The liver IAP transcripts mainly originate from a single IAP locus suggesting that the local chromatin environment or *cis*-acting mutations allow this copy of IAP to be expressed in the liver [50]. In contrast, the endogenous IAP transcripts present in pre-implantation embryos arise from at least two different IAP subtypes that have distinct expression patterns at these stages [35]. Sequences outside the LTR region might therefore be important for activating IAP expression in pre-implantation embryos.

### LINE-1 retrotransposon expression during germ cell development

**Fig. 3:**
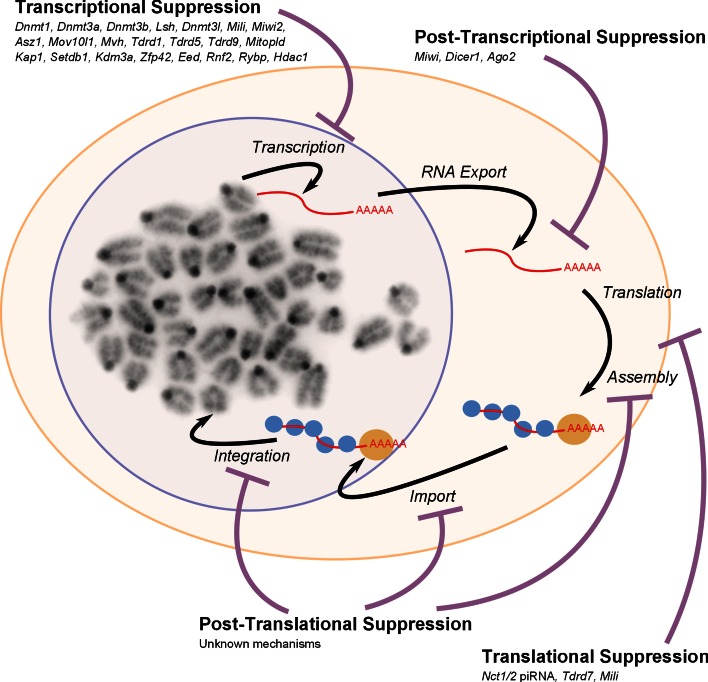
Retrotransposon suppression mechanisms. A schematic overview of a generalised retrotransposon life cycle encompassing transcription, RNA export, translation, assembly, nuclear import, and de novo integration is shown. Specific elements can show some variation in this process, e.g. RNA from non-autonomous SINE retrotransposons is not translated, and uses LINE-1-encoded proteins to catalyse their integration. Also, the mechanism of integration differs between LINE-1 and LTR retrotransposons. Genes involved in suppressing retrotransposons at specific stages of their life cycle in germ cells and pluripotent cells are indicated

The non-LTR LINE-1 retrotransposon has also been shown to be expressed at specific points in germ cell development. LINE-1 RNA, protein, ribonucleoprotein particles and de novo retrotransposition events are all detectable in pluripotent stem cell lines, and LINE-1 expression is downregulated when these cells differentiate into somatic cells [51–53]. A number of widely-expressed transcription factors including RUNX3 and YY1 have been implicated in activating human and/or mouse LINE-1 transcription [8, 54, 55]; however, more restricted transcription factors such as SOX2 could potentially confer some tissue and stage specificity [56]. LINE-1 RNA is also present in E11.5–E13.5 primordial germ cells that have colonised the genital ridge [39, 57]. However, LINE-1 ORF1 protein is not detectable in the germ cells until E15.5, a stage of development when male germ cells are quiescent and female germ cells are in leptotene/zygotene stages of meiotic prophase [58]. Interestingly, LINE-1 ORF1 protein levels decrease in germ cells after birth, but full-length LINE-1 transcripts and ORF1 protein peak again as male spermatocytes pass through the leptotene/zygotene stages of meiotic prophase [59]. Thus, early meiotic prophase may be a common point in male and female germline development for susceptibility to LINE-1 expression. LINE-1 ORF1 protein does not appear to be as abundant during later meiotic stages in either male or female germ cells [58, 59]. Interestingly, despite the high levels of LINE-1 expression during specific stages of male and female germline development, LINE-1 retrotransposition has been proposed to occur mainly in early pre-implantation embryos after fertilisation [60]. The poor correlation between LINE-1 RNA expression, LINE-1 protein expression and LINE-1 retrotransposition at some stages of the germline cycle could represent expression of non-functional full-length copies of LINE-1, or read-through transcription from adjacent host promoters into non-functional 5′-truncated copies of LINE-1. In addition, genome defence mechanisms could be acting to inhibit different stages of the LINE-1 life cycle (Fig. 3) and dampen down the retrotransposition potential of these elements.

### Retrotransposon activity in somatic tissues

Any de novo retrotransposition occurring in pluripotent cells can potentially generate genetic variation in the somatic and germline components of an individual (Fig. 2). Somatic variation has been observed for de novo LINE-1 integration events in neurons, and this genetic heterogeneity has been proposed to be important for brain function [56, 61–63]. Although de novo LINE-1 integration events in neurons could arise from LINE-1 activity in pluripotent cells [60], LINE-1 expression within the neuronal lineage [56] is also likely to contribute to this phenomenon. Although successful retrotransposons need to be active in pluripotent cells or the developing germline, there is also some retrotransposon expression in somatic tissues. Analysis of LTR retrotransposon expression across multiple tissues using retrotransposon-specific microarrays suggests that different LTR retrotransposons have different somatic expression profiles [64, 65]. However, some of this somatic tissue-specific expression could represent the behaviour of a small number of anomalous copies of any particular retrotransposon, rather than an inherent tissue-specificity of the LTR [50, 66]. One somatic tissue that has long been associated with retrotransposon expression is the placenta [67]. The trophectoderm-derived cells in the placenta have low levels of DNA methylation (Fig. 2), and their specialised epigenetic state is thought to make these cells permissive for expression of some retrotransposons [68, 69]. Specific subsets of LTR retrotransposons, including ERVWE1 elements in human and IAP elements in mouse [64, 69], are highly expressed in the placenta, and retrotransposon-encoded proteins and LTRs have been co-opted to perform host functions during placenta development [33, 70, 71]. Although retrotransposon expression or activity can be beneficial to the somatic tissues of their mammalian hosts, these elements have also been proposed to drive genetic instability and cancer [3, 19, 72]. Establishing mitotically heritable suppression of retrotransposons in the pluripotent cells early in development can therefore help to maintain genomic stability in the somatic tissues.

## Epigenetic silencing of retrotransposons by DNA methylation

One of the major mechanisms that mammalian cells use to defend their genomes against retrotransposons is transcriptional silencing (Fig. 3; Table 1). The main transcriptional silencing mechanisms known to be operating in mammalian cells typically involve adding and removing covalent modifications to DNA and histones [73]. These modifications can alter chromatin structure, and change the physical associations between proteins and the underlying DNA sequence. One type of modification that is truly epigenetic, in that it can mediate heritable changes in gene activity without changing DNA sequence, is DNA methylation [74].

**Table 1:** Repression mechanisms affecting different types of retrotransposon in mouse ES cells and germ cells

Retrotransposon family	Retrotransposon class	Enriched chromatin modifications (ES cells)	Repression mechanisms (ES cells)	Repression mechanisms (germ cells)
LINE-1	LINE	5mC	TRIM28, KDM1A	DNA methyltransferases, PIWI-piRNA pathway, TDRD7
ERV1	LTR	5mC, H3K9Me3, H4K20Me3	TRIM28, SETDB1, ZFP809, DNA methyltransferases, polycomb repressive complexes	Unknown
ERVK	LTR	5mC, H3K9Me3, H4K20Me3	TRIM28, SETDB1, HDAC1, DNA methyltransferases, polycomb repressive complexes	DNA methyltransferases, PIWI-piRNA pathway, LSH, TEX19.1, endogenous siRNA
ERVL	LTR	5mC, H3K27Me3	TRIM28, KDM1A, ZFP42, RYBP	Unknown
MaLR	LTR	5mC, H3K27Me3	TRIM28	Endogenous siRNA

Summary of some of the main mechanisms that have been shown to repress retrotransposons in mouse ES cells and germ cells. Different types of retrotransposon are repressed by different mechanisms, and are shown grouped by retrotransposon family

### DNA methylation in mammalian genomes

In mammals, DNA methylation occurs almost exclusively on cytosine residues in the context of CpG dinucleotides [74]. Most of the mammalian genome, including repetitive DNA sequences, is heavily methylated [75, 76], but this bulk methylation is interrupted by short stretches of DNA containing high densities of CpGs known as CpG islands (CGIs) that escape DNA methylation [77]. The density of CpGs in the CGI affects the methylation status of these sequences, which are often found coincident with gene promoters. High density CpG CGI promoters are almost always unmethylated and transcriptionally active except in rare tissue-specific genes and some tumour suppressor genes in cancer cells [75, 76, 78]. Intermediate density CpG CGI promoters are rarer than high density CpG CGI promoters but are more likely to be silenced and methylated in a tissue-specific manner [76]. In contrast, low CpG density CGIs promoters tend to be methylated irrespective of their transcriptional status [76]. The correlation between DNA methylation and transcriptional repression is therefore strongly influenced by CpG density and genomic context.

### Adding DNA methylation to the genome

Mammals possess three DNA methyltransferases that can catalyse methylation of CpG dinucleotides: DNMT1, DNMT3A and DNMT3B. DNMT1 perpetuates existing DNA methylation marks by methylating hemi-methylated DNA templates [79–81]. DNMT1 is thought to be recruited to sites of DNA replication by its protein partner PCNA [82], where it uses the parental strand as a methylation template to modify the newly synthesised daughter strand, thereby maintaining DNA methylation patterns during proliferation. A second protein, Np95 (also known as UHRF1), helps recruit DNMT1 to hemi-methylated CpGs generated at the replication fork during DNA replication [83, 84]. Np95 binding to heterochromatin-associated histone modifications during DNA replication is essential for the recruitment of this maintenance methyltransferase complex; this ensures that DNMT1 activity is stabilised when in a complex with other heterochromatin-associated proteins during the late phase of DNA replication to perpetuate a global methylation profile [84, 85]. During early development and gametogenesis, tissue-specific DNA methylation patterns are established in part by the two de novo DNA methyltransferases DNMT3A and DNMT3B [86]. *Dnmt3a* ^−*/*−^ *Dnmt3b* ^−*/*−^ double-knockout embryos have hypomethylated DNA, start to exhibit developmental retardation by E8.5, and die by E11.5 [86]. Interestingly, the de novo DNA methyltransferases have non-overlapping roles at some genomic sequences: DNMT3B, but not DNMT3A, has a key role in regulating methylation at centromeric minor satellite repeats, and both interspersed C-type endogenous retroviruses (which includes MuLV ERV1 LTR retrotransposons) and IAP elements are slightly hypomethylated in *Dnmt3b* ^−*/*−^ embryos but retain methylation in *Dnmt3a* ^−*/*−^ embryos [86]. IAP hypomethylation is more pronounced in embryos lacking both DNMT3A and DNMT3B, suggesting a degree of redundancy between these two enzymes at these loci [86]. Detailed analysis of DNA methylation differences between these knockout embryos using current genome-wide approaches might provide some insight into the differences in specificity between the de novo DNA methyltransferases.

Like the *Dnmt3a* ^−*/*−^ *Dnmt3b* ^−*/*−^ double mutants, embryos that carry hypomorphic mutant alleles of *Dnmt1* become developmentally retarded soon after gastrulation, and die by E12.5 [87]. Hypomethylation at IAP elements and C-type endogenous retroviruses, and increased expression of IAP retrotransposon transcripts, have all been shown to occur in these hypomorphic *Dnmt1* mouse embryos [45, 87]. DNMT1 can act as a transcriptional repressor if it is recruited to unmethylated DNA by protein-interacting partners, and DNA methylation-independent transcriptional repression by DNMT1 has been demonstrated in frogs and mammals [88, 89]. However, developmental retardation, embryonic lethality and IAP and LINE-1 retrotransposon hypomethylation all occur in embryos that carry catalytically inactive mutant alleles of *Dnmt1* [90], suggesting that these aspects of the *Dnmt1* mutant phenotype are a consequence of a failure to maintain DNA methylation.

### Removing DNA methylation from the genome

The molecular pathways governing the removal of DNA methylation from mammalian genomes are also starting to be more fully realised, and the TET family of dioxygenases are central to this process [91–94]. The TET enzymes can iteratively oxidise 5mC to generate 5-hydroxymethyl cytosine (5hmC), 5-formyl cytosine (5fC), and 5-carboxyl cytosine (5caC) [95]. 5hmC, 5fC and 5caC are not maintained by DNMT1 during DNA replication, and can therefore lead to demethylation as they are diluted during cell proliferation [96, 97]. Alternatively, the DNA repair machinery can excise 5fC and 5caC, or deaminated derivatives of modified cytosine residues, to bring about demethylation [98, 99]. 5hmC is less abundant than 5mC in the genome and is typically enriched in the bodies of actively transcribed genes, and at active enhancers and sequences surrounding transcriptional start sites [100–102]. The distribution of 5hmC across the genome is distinct between tissues, and 5hmC patterns can be used as an identifier of cell type or disease state [103, 104]. 5fC and 5caC are even less abundant than 5hmC, and can also be detected at some enhancers and gene regulatory elements, particularly when components of the DNA repair machinery are mutated to allow these transient intermediates to accumulate [105, 106].

Mammals possess three TET enzymes. *Tet1* ^−*/*−^ and *Tet2* ^−*/*−^ mice each exhibit some phenotypic abnormalities, but these mice are viable and fertile [107–110]. In contrast, *Tet3* is essential for viability and *Tet3* ^−*/*−^ mice die perinatally [111]. There is redundancy between TET1 and TET2 as *Tet1* ^−*/*−^ *Tet2* ^−*/*−^ double-knockout mice show some lethality at both embryonic and perinatal stages [112]. However, some *Tet1* ^−*/*−^ *Tet2* ^−*/*−^ double-knockout mice are viable and, aside from small ovaries, have no overt phenotypic abnormalities [112]. *Tet1* ^−*/*−^ *Tet2* ^−*/*−^ double-knockout mice have reduced levels of 5hmC and increased levels of 5mC in somatic tissues, consistent with these genes having a role in converting 5mC to 5hmC [112]. TET enzymes play a role in reprogramming methylation patterns at some sequences during development, and are contributing to the general dynamic reprogramming of DNA methylation in the genome, which can possibly include retrotransposon sequences [105, 106, 110–113].

### DNA methylation and transcriptional repression

DNA methylation is strongly enriched at retrotransposon sequences in mammalian genomes, so much so that the primary role of DNA methylation has been proposed to be transcriptional repression of these elements [114]. DNA methylation could exert its repressive effects on transcription by sterically interfering with transcription factors binding to their cognate binding sites in promoter proximal regulatory regions [115]. Alternatively, a number of methyl-CpG-binding proteins have been identified in mammals that could influence transcription factor accessibility or chromatin structure at genomic sites containing this epigenetic mark [116–119]. For example, the methyl-CpG-binding protein MECP2 is thought to mediate transcriptional repression of methylated DNA at least partly through its interaction with the SIN3A co-repressor complex [120]. It is not yet clear if methyl-CpG-binding proteins are necessary for global silencing of methylated target genes or retrotransposons in mammalian cells [121]. However, the strong correlation between DNA methylation and transcriptional repression in the genome [76] primarily represents DNA methylation acting to ‘lock-down’ transcription after a repressed state has already been established by other mechanisms, and only a small number of mammalian genes appear to rely on DNA methylation as a primary mechanism to silence their expression [43].

Experimentally removing DNA methylation in mouse somatic tissues strongly induces expression of IAP retrotransposons, suggesting that DNA methylation has a primary role in repressing these elements in mouse embryonic fibroblasts [42–44]. It is not clear how many other retrotransposons rely predominantly on DNA methylation for their repression in somatic tissues. MMVL30 and MuLV elements, which are both members of the ERV1 family of LTR retrotransposons, are transcriptionally upregulated in response to DNA hypomethylation in mouse embryonic fibroblasts, suggesting that DNA methylation might be a primary transcriptional silencing mechanism for these elements [122]. However, recent data, showing that re-distribution of polycomb-associated histone modifications is responsible for around a third of the transcriptional changes that occur in hypomethylated fibroblasts [123], illustrate the complex interactions that exist between different chromatin modifying mechanisms and the difficulties associated with interpreting these data mechanistically. Notably, both MMVL30 and MuLV elements are upregulated in ES cells carrying mutations in components of polycomb repressive complexes [124, 125].

Like the LTR retrotransposons, non-LTR LINE and SINE retrotransposons are also highly methylated in somatic tissues [126], but transcriptional silencing of these elements does not strongly depend on DNA methylation in fibroblasts [122]. DNA methylation and MECP2 have been implicated in repressing mouse and human LINE-1 retrotransposons in neuronal cells [63]. However, MECP2 is a global regulator of neuronal chromatin structure, and the modest increase in retrotransposon expression seen in *Mecp2* ^−*/*−^ brains could reflect an increase in transcriptional noise [127].

## Transcriptional silencing of retrotransposon expression in embryonic stem cells

Although a number of studies have reported that loss of DNA methylation causes strong upregulation of IAP elements in somatic tissues, ES cells carrying mutations in all three DNA methyltransferases exhibit a more modest upregulation of these elements, even though IAP DNA methylation is severely reduced [42–45, 125, 128–130]. Therefore, DNA methylation does not play as dominant a role in IAP suppression in ES cells as it does in somatic cells. Microarray and deep-sequencing analysis suggests that a relatively small number of LTR retrotransposons are upregulated in the hypomethylated *Dnmt1* ^−*/*−^ *Dnmt3* ^−*/*−^ *Dnmt3b* ^−*/*−^ triple-knockout (*Dnmt* ^*TKO*^) ES cells [125, 129]. Notably, LINE-1 elements and MuLV elements, which have been reported to be regulated by DNA methylation in neuronal and fibroblast somatic cell types, respectively [63, 122], are not strongly de-repressed in *Dnmt* ^*TKO*^ ES cells despite being hypomethylated [129, 130]. In contrast, LTR retrotransposons belonging to ERV1 (MMERGLN, RLTR1B) and ERVK (IAP, RLTR45, MMERVK10C, RMER16) families are all upregulated in *Dnmt* ^*TKO*^ ES cells [129]. The level of upregulation for each of these elements (~2.3- to 13-fold) is relatively modest, suggesting that additional retrotransposon silencing mechanisms might be operating in ES cells.

An alternative repressive modification that is associated with retrotransposons in ES cells is histone 3 lysine 9 trimethylation (H3K9Me3) [131]. H3K9Me3 is highly enriched on ERV1 and ERVK families of LTR retrotransposons in ES cells, but not on the ERVL and MaLR families [131]. ES cells that carry mutations in the SETDB1 (also known as ESET) histone methyltransferase have reduced levels of H3K9Me3 at LTR retrotransposon sequences, and a significant de-repression of many different types of LTR retrotransposon [125, 129, 132]. In contrast to the *Dnmt* ^*TKO*^ ES cells, 69 different LTR retrotransposons are de-repressed in *Setdb1* ^−*/*−^ ES cells [129]. The upregulated LTR retrotransposons primarily belong to the ERV1 and ERVK families, whereas ERVL and LINE-1 retrotransposons are not strongly upregulated in *Setdb1* ^−*/*−^ ES cells [129]. Interestingly, each LTR retrotransposon that is upregulated in *Dnmt* ^*TKO*^ ES cells is more strongly upregulated in *Setdb1* ^−*/*−^ ES cells. Furthermore, the LTR retrotransposon upregulation in *Dnmt* ^*TKO*^ ES cells is not associated with decreased levels of H3K9Me3 at these elements, and DNA methylation at these elements is not strongly affected in *Setdb1* ^−*/*−^ ES cells, suggesting that these silencing mechanisms are recruited independently, and function in parallel, to repress transcription of these elements [129]. Although H3K9Me3 is enriched on ERV1 and ERVK LTR retrotransposons in fibroblasts, as it is in ES cells [131], a clear difference exists between these cell types in their requirement for SETDB1 in this process. Whereas SETDB1 is bound to ERV1 and ERVK retrotransposons and mediates H3K9Me3 and repression at these loci in ES cells, SETDB1 is neither bound at these sequences nor required for their H3K9Me3 in fibroblasts [129, 132]. Thus, SETDB1-dependent H3K9Me3 appears to play an important role in repressing ERV1 and ERVK retrotransposons in ES cells, but not in somatic fibroblasts.

Transcriptional silencing of the ERVL family of LTR retrotransposons in ES cells does not appear to depend on SETDB1 [129], but relies instead on the histone demethylase KDM1A [133]. *Kdm1a* ^−*/*−^ ES cells de-repress MERVL elements, LINE-1 elements and a number of chimaeric transcripts containing endogenous genes driven from ERVL and MaLR promoters similar to those found in zygotes [133]. ERVL and MaLR retrotransposon DNA is highly methylated in ES cells and somatic tissues [39, 126], but DNA methylation at these elements is not perturbed in *Kdm1a* ^−*/*−^ ES cells [133]. Rather, loss of KDM1A results in a number of changes to the histone modifications present at ERVL and MaLR LTRs in ES cells, with histone 3 lysine 4 methylation (H3K4Me) and histone 3 lysine 27 acetylation (H3K27Ac) levels both increasing and H3K9Me3 levels decreasing [133]. KDM1A physically interacts with the HDAC family of histone deacetylases, and the increase in H3K4Me and H3K27Ac levels at target retrotransposons in *Kdm1a* ^−*/*−^ ES cells might reflect reduced demethylase (KDM1A) and deacetylase (HDAC) activities, respectively, at these sequences [133]. ERVL and MaLR retrotransposons are enriched for the polycomb-associated histone 3 lysine 27 trimethylation (H3K27Me3) modification in wild-type ES cells [131], but H3K27Ac will prevent acquisition of the repressive trimethyl modification at the same residue. ES cells carrying mutations in components of the polycomb repressive complexes de-repress various different retrotransposons, including ERV1 (MuLV, MMVL30) and ERVK (IAP, RLTR45) LTR retrotransposons [124, 125]. However, ERVL and MaLR LTR retrotransposons are not strongly de-repressed in these mutant ES cells, despite being strongly enriched for the H3K27Me3 mark [124, 125, 131]. Thus, the de-repression of ERVL elements and MaLR sequences in *Kdm1a* ^−*/*−^ probably reflects multiple changes in histone modification and chromatin structure at these sequences. The *Kdm1a*-dependent repression of ERVL and MaLR retrotransposons seems to be associated with the zinc finger protein ZFP42 (also known as REX1). ZFP42 physically interacts with KDM1A and is bound to ERVL and MaLR retrotransposon DNA in ES cells [134]. Like *Kdm1a* ^−*/*−^ ES cells, *Zfp42* ^−*/*−^ ES cells also de-repress MERVL retrotransposons [134]. Thus, ZFP42 could potentially provide sequence specificity to this silencing mechanism and recruit KDM1A to MERVL and MaLR retrotransposon DNA in order to transcriptionally repress these elements. RYBP, a protein that interacts with ZFP42, also plays a role in repressing MERVL retrotransposons in ES cells [135].

Although there are differences in the mechanisms repressing the different LTR retrotransposon families (summarised in Table 1), one factor that has strong effects across the entire class of LTR retrotransposons is TRIM28 (also known as KAP1). TRIM28 plays a role in silencing multiple LTR retrotransposons in ES cells including MuLV, IAP, MERVL and MT elements belonging to each of the ERV1, ERVK, ERVL and MaLR families [136, 137]. *Trim28* ^−*/*−^ ES cells also modestly upregulate LINE-1 retrotransposons [136]. TRIM28 can be recruited to specific DNA sequences via its interactions with Krüppel-associated box zinc finger proteins, and has been shown to interact with repressive chromatin-modifying enzymes such as SETDB1 and KDM1A [133, 138]. In ES cells, TRIM28 is recruited to a specific sequence in MuLV retrotransposons by the zinc finger protein ZFP809 [137, 139]. The sequences responsible for TRIM28-dependent repression of MuLV are distinct from the sequences implicated in TRIM28-dependent repression of IAP elements, suggesting that different DNA-binding proteins are probably involved in recruiting TRIM28 to different retrotransposons [136]. *Trim28* ^−*/*−^ ES cells have reduced levels of H3K9Me3 at IAP elements, which presumably reflects impaired recruitment of SETDB1 to these loci, and upregulate IAP transcription around 20-fold [129, 132, 136, 138]. Notably, DNA methylation at IAP elements is not strongly affected in *Trim28* ^−*/*−^ ES cells [136, 140], suggesting that TRIM28 is silencing retrotransposons independently of DNA methylation in this cell type. Interestingly, the primary transcriptional silencing mechanism for IAP retrotransposons appears to switch from being TRIM28/SETDB1-dependent histone modification in ES cells to DNA methylation in somatic cells [42, 43, 45, 128, 129, 132, 136]. Recent findings that differentiation of pluripotent *Trim28* ^−*/*−^ cells into somatic cells results in some reduction in the amount of DNA methylation at IAP retrotransposons suggests that there is a temporal link between TRIM28 and DNA methylation during differentiation and development [140]. These results are consistent with the concept that DNA methylation is generally recruited to chromatin after a repressed state has been established to stably repress transcription during somatic differentiation.

## Transcriptional silencing of retrotransposons in the germline

Although a number of recent advances have been made in identifying transcriptional silencing mechanisms operating on retrotransposons in pluripotent cells, much less is known about the transcriptional regulation of these elements in developing germ cells (Table 1). DNA methylation and many histone modifications have been shown to undergo transient global decreases at multiple points in the germline cycle [141–144]. Genome-wide analysis of the sequences associated with specific histone modifications during germ cell development is starting to become technically possible [145], and it will be of interest to elucidate whether some of the transcriptional silencing mechanisms operating on retrotransposons in ES cells are also important for retrotransposon silencing in germ cells. However, at present, the best-studied transcriptional silencing mechanism operating at retrotransposon sequences in germ cells is DNA methylation.

### DNA methylation in the developing germline

DNA methylation patterns undergo dynamic changes during germ cell development (Fig. 2). The global level of DNA methylation in primordial germ cells decreases progressively between E8.5 and E12.5, although different sequences exhibit different dynamics during this process [39, 46, 146]. Genome-wide bisulfite sequencing shows that LINE retrotransposons, SINE retrotransposons and ERV1, ERVK, ERVL and MaLR LTR retrotransposons are all hypomethylated in E13.5 germ cells, and typically only have around 20 % of their CpG’s methylated compared to around 80 % in foetal somatic tissues [126]. Some LTR retrotransposons, such as IAP elements, are more resistant to this hypomethylation event than others and still retain an intermediate amount of DNA methylation (~60 %) in E13.5 germ cells [39, 147, 148]. Multiple intersecting pathways are likely to be involved in mediating this global demethylation, and conversion of 5mC to 5hmC by TET1 and TET2 enzymes [113], deamination of 5mC by AID (also known as AICDA) [126], passive loss of cytosine modifications during DNA replication [39, 113, 149], active removal of nucleotides by the DNA repair machinery [150] and downregulation of DNA methyltransferases and their accessory factors [39, 46, 151] are all implicated in this process.

Once the germ cells have undergone global hypomethylation, DNA methylation patterns are re-established on retrotransposon sequences in a sex-specific manner (Fig. 2). Male germ cells undergo de novo methylation during late foetal and early postnatal development [152], long before male germ cells initiate meiosis. Genome-wide bisulfite sequencing shows that de novo methylation in the male germline generates sperm with similar, or even slightly higher, genome-wide and retrotransposon methylation levels to foetal somatic tissues [39]. In contrast, de novo methylation in female germ cells occurs in postnatal growing oocytes, after the oocytes have completed the early stages of meiotic prophase and are held in a dictyate stage meiotic arrest [153, 154]. De novo methylation increases the amount of DNA methylation in postnatal oocytes, but mature eggs remain hypomethylated relative to foetal somatic tissues and sperm [154, 155]. Although LINE, SINE, and LTR retrotransposons are all de novo methylated during oocyte growth, some copies of these retrotransposons remain partially unmethylated in mature eggs [154, 155]. Acquisition of de novo methylation at some imprinted genes and intragenic CGIs in oocytes is associated with transcription through these regions [154, 156], but it is not clear whether this association extends to retrotransposons.

After fertilisation, DNA methylation declines during pre-implantation development (Fig. 2), with LINE, SINE and LTR retrotransposon sequences all losing DNA methylation [155, 157, 158]. Some retrotransposons, including IAP elements, are somewhat resistant to this hypomethylation event [148, 158]. At the blastocyst stage of pre-implantation development, DNA methylation levels increase in the pluripotent epiblast cells [157], and genome-wide bisulfite sequencing of ES cells derived from this tissue shows that global and retrotransposon DNA methylation levels are similar between ES cells, post-implantation epiblast, and foetal somatic tissues [39, 126]. In contrast, DNA methylation levels remain low in the trophectoderm layer of the blastocyst [157], and the placenta has moderate levels of methylation (~40–50 %) at LINE, SINE and LTR retrotransposon sequences [126], representing a mixed population of hypomethylated trophectoderm-derived and hypermethylated epiblast-derived tissues.

### De novo DNA methylation of retrotransposon sequences during gametogenesis

Mutations that interfere with de novo DNA methylation might be expected to cause defects in epigenetic reprogramming and retrotransposon silencing in the developing germline. The lethality of *Dnmt3a* ^−*/*−^ and *Dnmt3b* ^−*/*−^ embryos has necessitated the generation of conditional mutants to investigate the function of these genes in the germline [159]. *Dnmt3a* ^−*/*−^ and *Dnmt3b* ^−*/*−^ prospermatogonia have normal DNA methylation levels at IAP and LINE-1 elements, possibly indicating functional redundancy between DNMT3A and DNMT3B at these loci [152, 159, 160]. In contrast, de novo methylation of B1 SINE retrotransposons in spermatogonia requires DNMT3A but not DNMT3B [152]. In oocytes, there is redundancy between DNMT3A and DNMT3B for de novo DNA methylation at IAP elements, but only DNMT3A is involved in de novo DNA methylation of LINE-1 elements [161]. Thus, the de novo methyltransferases have different specificities for retrotransposon sequences.

The de novo methyltransferase activity of both DNMT3A and DNMT3B is stimulated by the catalytically inactive DNA methyltransferase-like protein DNMT3L [162]. In contrast to *Dnmt3a* and *Dnmt3b*, *Dnmt3l* is not required for viability or retrotransposon DNA methylation in somatic tissues. However, *Dnmt3l* ^−*/*−^ mice are infertile and have defects in de novo methylation during spermatogenesis and oogenesis [152, 163–166]. *Dnmt3l* ^−*/*−^ prospermatogonia and oocytes have reduced levels of DNA methylation at LINE-1, IAP and B1 SINE retrotransposons [152, 154, 155, 161, 163, 166]. The hypomethylation at IAP and LINE-1 retrotransposons in *Dnmt3l* ^−*/*−^ prospermatogonia is associated with transcriptional activation of these elements in foetal and perinatal testes [163]. De novo methylation of IAP and LINE-1 retrotransposons in foetal male germ cells therefore seems to be required for transcriptional silencing of these elements in the prospermatogonia.

Another factor that has been linked with de novo DNA methylation of retrotransposons sequences and genome defence is LSH (also known as HELLS). LSH is not restricted to the germline and is required for normal embryonic DNA methylation of repetitive elements and some single copy genes in somatic tissues [167, 168]. LSH has been proposed to recruit the de novo DNA methyltransferase DNMT3B to specific target sites in the genome [169]. Notably, loss of LSH function is sufficient to de-repress IAP transcription in mouse embryonic fibroblasts, alter pericentromeric heterochromatin, and induce gene expression changes at single copy genes [170, 171]. The DNA hypomethylation at IAP elements that occurs in *Lsh* ^−*/*−^ somatic tissue is also evident in *Lsh* ^−*/*−^ oocytes, and IAP expression is greatly increased in *Lsh* ^−*/*−^ foetal ovaries [172]. As IAP is normally hypomethylated in meiotic oocytes until the postnatal oocyte growth phase [153], either the IAP hypomethylation in *Lsh* ^−*/*−^ oocytes is affecting a subset of CpGs critical for transcriptional repression of these elements, or the transcriptional upregulation of IAP in *Lsh* ^−*/*−^ ovaries is a feature of the somatic cells in the ovary rather than oocytes. The role of *Lsh* in de novo DNA methylation in foetal prospermatogonia and growing oocytes has not yet been studied, and it will be of interest to determine whether de novo methylation events occurring at different stages of the germline cycle (Fig. 2) have different requirements to direct the DNA methylation machinery to target loci.

### PIWI proteins and retrotransposon DNA methylation

**Fig. 4:**
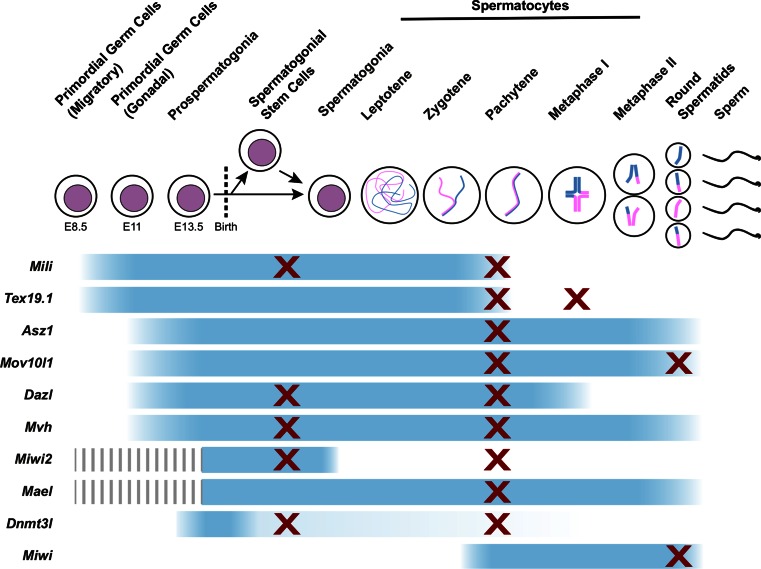
Expression patterns and mutant phenotypes of germline genome defence genes. The stages of spermatogenesis are indicated along the *top* of the diagram, and expression patterns of the indicated germline genome defence genes are indicated by *blue bars*. The stages at which mutant mice are reported to have defects in progression through spermatogenesis are indicated with *crosses*

A number of additional genes are also required for de novo methylation and transcriptional silencing of retrotransposons in male germ cells. PIWI proteins belong to a subclade of the Argonaute family of small RNA binding proteins and contain RNA binding and RNaseH-like endonuclease domains [173]. Mice possess three PIWI proteins: MIWI (also known as PIWIL1), MILI (also known as PIWIL2), and MIWI2 (also known as PIWIL4), that each physically interact with populations of germline-restricted small, single-stranded RNAs known as PIWI-interacting RNAs (piRNAs) [174–178]. Expression of the three PIWI proteins is largely restricted to the germline, but each PIWI protein has a different expression pattern during germ cell development (Fig. 4). *Mili* and *Miwi2* are expressed in late foetal male germ cells when de novo methylation of retrotransposon DNA occurs [176, 178]. *Mili* ^−*/*−^ and *Miwi2* ^−*/*−^ prospermatogonia fail to de novo methylate IAP and LINE-1 elements, and de-repress transcription of these elements [47, 175–177]. It is not clear whether the methylation state of other retrotransposons is affected in these mutant germ cells, and genome-wide analysis of their DNA methylation patterns would be of interest in this regard.

Although *Mili* and *Miwi2* are required genetically for the de novo methylation of retrotransposons [47, 175–177], the mechanistic role that PIWI proteins play in this process is not fully understood. MILI and MIWI2 physically interact with piRNAs that originate from genomic piRNA clusters, retrotransposons and other genomic regions [47, 175, 176]. Ping-pong amplification of piRNA sequences occurs when an antisense piRNAs guides the cleavage of a complementary mRNA to generate sense piRNAs, which in turn can guide cleavage of complementary sequences in piRNA cluster transcripts to generate more antisense piRNAs to feed back into the system. Molecular chaperones are thought to be important to dissociate PIWI-piRNA complexes, allowing ping-pong amplification to occur [179]. MILI and MIWI2 physically associate with different subpopulations of piRNA in foetal prospermatogonia, and have different roles in de novo methylation of retrotransposon sequences [47, 176]. The putative endonuclease activity of MILI appears to be required for ping-pong amplification of retrotransposon piRNAs in foetal prospermatogonia, and de novo methylation and transcriptional silencing of LINE-1, but not IAP elements [180]. In contrast, the putative endonuclease activity of MIWI2 is not required for these events [180], and MIWI2 has been proposed to function by translocating into the nucleus when loaded with piRNA, where it uses the piRNA sequence as a guide to recruit the de novo methylation machinery to actively transcribing retrotransposons [176]. However, physical interactions between MIWI2 and either DNMT3A or DNMT3B have not been detected in foetal prospermatogonia [176]. It is possible that the MIWI2/piRNA complexes recruit the de novo DNA methylation machinery indirectly, perhaps through inducing repressive histone modifications at retrotransposon loci. However, although there is strong genetic evidence that *Mili* and *Miwi2* are required for de novo DNA methylation of LINE-1 and IAP retrotransposons in male germ cells, the requirement for piRNA in this phenomenon is yet to be formally demonstrated. In this respect, it is notable that the third mouse PIWI protein, MIWI, has recently been shown to have some piRNA-independent functions in regulating gene expression [181].

De novo methylation of retrotransposons in foetal prospermatogonia also requires a number of additional factors that act in the PIWI–piRNA pathway. Tudor domain-containing proteins physically interact with methylated arginine residues in PIWI proteins [182–184], and co-localise with PIWI proteins and piRNAs in electron-dense cytoplasmic structures termed nuage [185–188]. The formation of these structures and interactions between them are thought to be crucial for the biogenesis of piRNAs and functional piRNA–PIWI complexes, and TDRD1, TDRD5 and TDRD9 are all required for normal DNA methylation of LINE-1 retrotransposons [186, 187, 189]. *Tdrd1* ^−*/*−^, *Tdrd5* ^−*/*−^ and *Tdrd9* ^−*/*−^ mutations all have stronger effects on transcriptional silencing of LINE-1 than IAP retrotransposons [186, 187, 189], again suggesting that there are differences in the requirements for de novo methylation of these elements.

Additional nuage-associated factors, including MITOPLD (also known as PLD6), MOV10L1, MVH (also known as DDX4), MAEL, and ASZ1 (also known as GASZ) are all required for DNA methylation and transcriptional silencing of LINE-1 retrotransposons through their involvement in the piRNA–PIWI pathway [190–194]. MITOPLD and MOV10L1 appear to be required for piRNA biogenesis [192, 193], and MVH is reported to be involved in piRNA processing [194]. MAEL does not appear to be required for piRNA biogenesis, but is speculated to play a role in assembly of PIWI–piRNA complexes or shuttling PIWI–piRNA complexes to or from the nuage [191], whereas ASZ1 is required to stabilise MILI [190].

Although each of the three mouse PIWI proteins is expressed in male germ cells and required for spermatogenesis, only MILI is expressed in the female germline [176–178, 195, 196]. MILI is not required for oogenesis but does appear to play a role in retrotransposon suppression in oocytes [197]. The levels of IAP transcript are modestly upregulated 3.5-fold in *Mili* ^−*/*−^ oocytes, but, in contrast to *Mili* ^−*/*−^ male germ cells, LINE-1 transcript levels are not affected. The level of IAP upregulation in *Mili* ^−*/*−^ oocytes is around 10-fold lower than the level of upregulation in *Mili* ^−*/*−^ testes [175], and it remains to be determined if the increase in IAP expression in *Mili* ^−/−^ mutant oocytes is due to defects in de novo methylation at these elements, or is a result of MILI’s proposed additional roles in post-transcriptional regulation [47, 198].

## Post-transcriptional suppression of retrotransposon RNAs in the germline

There is some evidence that retrotransposon RNAs can be suppressed at a post-transcriptional level in germ cells (Fig. 3). The putative endonuclease activity of PIWI proteins could potentially allow PIWI–piRNA complexes to destabilise retrotransposon RNAs via endonucleolytic cleavage. This ‘slicer’ activity towards retrotransposon mRNAs has recently been demonstrated for MIWI [199]. MIWI is expressed postnatally from the zygotene stage of meiosis until the haploid round spermatid stage [195], although the piRNA populations in post-pachytene male germ cells show little evidence of ping-pong amplification [181, 200]. MIWI-associated piRNAs are able to guide MIWI to cleave target RNAs with extensive complementarity [199]. LINE-1 RNA is a target for MIWI slicer activity and LINE-1 transcript levels are increased ~10-fold in round spermatids carrying a catalytically inactive allele of *Miwi* that lacks endonuclease activity [199]. LINE-1 DNA methylation and repeat-derived piRNA abundance are not altered in the *Miwi* endonuclease mutant round spermatids; however, the likely post-transcriptional increase in LINE-1 transcripts in these mutants could represent a combination of piRNA-dependent and piRNA-independent functions of MIWI [199, 201]. It is not clear if MIWI’s slicer activity is also important for post-transcriptional suppression of other types of retrotransposon, but this activity is likely to act as a back-up genome defence mechanism to target LINE-1 retrotransposons that have escaped suppression by transcriptional silencing mechanisms.

Post-transcriptional suppression of retrotransposons also occurs in female germ cells. Endogenous double-stranded small interfering RNAs (siRNAs) are present in oocytes, where they play a role in post-transcriptional silencing of retrotransposons [197, 202, 203]. Production of these endogenous siRNAs requires DICER1, an endoribonuclease that is expressed at high levels throughout oocyte growth [197]. The endogenous siRNAs in oocytes physically associate with AGO2 [197], an Argonaute protein that forms the catalytic component of the RNA-induced silencing complex [204]. Members of the ERVK and MaLR families of LTR retrotransposons (RLTR10 and MTA elements, respectively) are upregulated ~3- to 5-fold in *Dicer1* ^−*/*−^ and *Ago2* ^−*/*−^ growing oocytes, but LINE-1 and IAP transcript abundance does not change [197, 203]. Interestingly, whereas *Dicer1*/*Ago2* suppress MTA and RLTR10 but not IAP retrotransposons in growing oocytes, *Mili* suppresses IAP but not MTA or RLTR10 retrotransposons in these cells [197]. Thus, the *Mili*/piRNA and *Dicer1*/*Ago2*/endogenous siRNA pathways are associated with silencing different retrotransposons in growing oocytes.

Interestingly, LINE-1 expression in growing oocytes is not affected by any of the *Mili* ^−*/*−^, *Dicer1* ^−*/*−^ or *Ago2* ^−*/*−^ mutations [197]. However, post-transcriptional silencing of LINE-1 retrotransposons in the fully-grown oocytes depends on the putative RNA-binding protein MARF1 [205]. *Marf1* ^−*/*−^ oocytes exhibit numerous changes in mRNA abundance, including an upregulation in the levels of LINE-1 and IAP transcripts, despite there being no significant changes in IAP or LINE-1 DNA methylation [205]. It will be of interest to determine whether MARF1 directly interacts with LINE-1, IAP and any other retrotransposon mRNA in oocytes, and how any sequence specificity is determined.

## Other mechanisms of retrotransposon suppression

Although germ cells possess multiple mechanisms to prevent the accumulation of retrotransposon transcripts (Fig. 3), retrotransposon RNAs are readily detectable and even abundant in germ cells and pluripotent cells in wild-type mice. Furthermore, discrepancies between full-length retrotransposon transcript abundance, protein abundance, and retrotransposition activity at different stages of the germline cycle suggest that additional mechanisms that suppress retrotransposons at later stages of their life cycles are operating in the germline [32, 35, 38, 40, 52, 59, 60]. The Tudor domain-containing protein TDRD7 plays a role in translational regulation of LINE-1 retrotransposons in male germ cells [206], and two of the PIWI proteins, MILI and MIWI, physically associate with actively translating mRNAs in polysomes and are implicated in translational regulation [198, 207]. Interestingly, some piRNAs are also physically associated with polysomes in mouse testes [207]. It is not known whether retrotransposon mRNAs are targets for translational regulation by MILI or MIWI, but there is evidence that piRNAs can suppress translation of LINE-1 mRNAs. Deletion of a genomic cluster of piRNAs (*Nct1/2*) that includes an antisense LINE-1 sequence results in a modest ~1.5-fold increase in LINE-1 mRNA, but a striking ~15-fold increase in levels of LINE-1-encoded ORF1 protein [208]. piRNA-containing protein complexes could suppress LINE-1 translation in a similar way that microRNAs suppress translation of their target mRNAs [204].

Genome defence mechanisms operating at even later stages of the retroviral life cycle have been demonstrated in somatic tissues. MOV10, a putative RNA helicase, physically associates with human LINE-1 ribonucleoprotein particles and mouse IAP retroviral-like particles, and inhibits retrotransposition of both these elements in cellular assays, and can affect multiple stages of the retroviral life cycle [209–211]. Interestingly, germ cells express a paralog of the somatic *Mov10* gene, *Mov10l1*. MOV10L1 physically interacts with MILI, MIWI2 and MIWI proteins, and *Mov10l1* ^−*/*−^ testes have reduced amounts of piRNA, hypomethylation of LINE-1 DNA and de-repression of LINE-1 and IAP elements [193, 212]. Conditional deletion of *Mov10l1* from the pachytene stage of spermatogenesis onwards reveals that MOV10L1 has additional functions in late spermatogenesis [201]. *Mov10l1* ^−*/*−^ round spermatids display elevated DNA damage, which could indicate increased retrotransposition in these cells. Although LINE-1 and IAP RNA levels are not elevated in *Mov10l1* ^−*/*−^ round spermatids [201], MOV10L1 could, like its somatic paralog, be suppressing LINE-1 and IAP activity at later stages of the retrotransposon life cycle.

Another protein that is operating to repress retrotransposons in the developing germline is TEX19.1, a predominantly cytoplasmic protein in germ cells and pluripotent cells that represses the ERVK LTR retrotransposon MMERVK10C in postnatal testes [213]. Unlike a number of genes implicated in germline genome defence, *Tex19.1* does not appear to have a strong effect on endogenous gene expression, and the largest changes in gene expression in *Tex19.1* ^−*/*−^ testes are retrotransposon transcripts [125, 213]. LINE-1, B1 SINE and IAP retrotransposons are not upregulated in *Tex19.1* ^−*/*−^ knockout testes, and microarray analysis suggests that retrotransposon upregulation in *Tex19.1* ^−*/*−^ testes is primarily restricted to MMERVK10C [125, 213]. The exact molecular mechanisms of how TEX19.1 suppresses accumulation of MMERVK10C transcripts is not yet known, but studies on the role of *Tex19.1* in somatic placenta tissue illustrate one of the complexities in determining retrotransposon targets for genome defence mechanisms: in contrast to *Tex19.1* ^−*/*−^ testes, multiple retrotransposons including MMVL30 LTR retrotransposons and LINE-1 elements are upregulated in *Tex19.1* ^−*/*−^ placenta [69]. The presence of multiple complementary genome defence mechanisms in the developing germline is likely to be masking some of the effects of mutating individual retrotransposon suppression mechanisms in these cells.

## Regulation of germline genome defence genes by DNA methylation

A number of genes involved in suppressing retrotransposons in the germline have been shown to be regulated by DNA methylation. *Dazl*, *Zfp42*, *Mvh* and *Mael* have all been identified as belonging to groups of genes that require *Dnmt3b*-dependent promoter DNA methylation to silence their expression [214, 215]. Furthermore, analysis of gene expression changes in multiple somatic cell models for hypomethylation identified a core subset of 26 methylation-sensitive genes, which is highly enriched for genes involved in suppressing retrotransposons in the germline [43]. This group of methylation-sensitive germline genome defence genes, which includes *Tex19.1*, *Mili*, *Dazl*, *Asz1* and *Mov10l1*, is strongly and heritably upregulated in response to DNA hypomethylation [43]. Interestingly, methylation-sensitive germline genome defence gene promoters are not enriched for commonly studied repressive histone modifications, such as H3K27Me3, H3K9Me3 and histone 4 lysine 20 trimethylation (H4K20Me3), when they are transcriptionally repressed in somatic cells [43]. The lack of enrichment for these repressive histone modifications could explain why silencing of these genes is so dependent on DNA methylation.

During germline development, expression of the methylation-sensitive germline genome defence genes is initiated during the global wave of demethylation that occurs progressively in primordial germ cells between E8.5 and E12.5 (Fig. 4) [43, 46, 146]. Hypomethylation and expression of *Tex19.1* and *Mili* initiates early during this reprogramming event at ~E8.5–E9.5 while the primordial germ cells are migrating [43]. Hypomethylation and initiation of *Dazl*, *Mov10l1* and *Asz1* expression occurs towards the end of this epigenetic reprogramming event at ~E10.5–E11.5, after the germ cells have colonised the gonad [43, 216]. It is not known whether the differences in the timing of hypomethylation at different germline genome defence gene promoters reflect recruitment of different demethylation machineries to these genes [98]. Generally, expression of the germline genome defence genes is maintained throughout foetal germ cell development (Fig. 4) and in oocytes in adult females. In males, postnatal expression of the germline genome defence genes starts to decline in pachytene spermatocytes (*Tex19.1*, *Mili* and *Dazl*) and in round spermatids (*Asz1* and *Mov10l1*) (Fig. 4) [43, 176, 178, 190, 193, 217–219]. It is not clear whether DNA methylation transcriptionally represses these genes in these later stages of spermatogenesis.

Global DNA hypomethylation in the primordial germ cells extends to retrotransposon sequences [39, 126, 147, 148], and removing this extra layer of repression could potentially lead to variant copies of these elements being transcribed. The activation of IAP LTR transgenes in male foetal germ cells from ~E16 [40] is consistent with the hypothesis that at least some copies of these methylation-sensitive retrotransposons are becoming transcriptionally de-repressed in hypomethylated germ cells. Indeed, it is possible that some transcriptional de-repression of retrotransposons is a pre-requisite for the generation of piRNA, and PIWI–piRNA-mediated targeting of de novo methylation to retrotransposon sequences [176]. Notably, germline genome defence genes that act at post-transcriptional stages of the retrotransposon life cycle (Fig. 3) could play an important role in limiting retrotransposon activity during these periods of global hypomethylation. Coupling expression of post-transcriptional genome defence mechanisms to the initiation of this epigenetic reprogramming process provides an effective way to ensure that appropriate genome defence mechanisms are active during the developmental window when the potential for retrotransposons to become active is high.

Hypomethylated cells in the mammalian placenta (Fig. 2) are another potential site for transcriptional de-repression of retrotransposons and activation of methylation-sensitive germline genome defence genes. Retrotransposons are hypomethylated in the placenta [68, 126, 220], but *Tex19.1* is the only one of the germline genome defence genes to be strongly hypomethylated and expressed at similar levels in the placenta and the testis [69]. LINE-1 retrotransposons are upregulated in the hypomethylated trophectoderm-derived component of *Tex19.1* ^−*/*−^ placentas, suggesting that this germline genome defence gene is functionally repressing retrotransposons in this hypomethylated somatic context [69]. Although the germline genome defence genes are normally methylated and silenced in embryo-derived somatic tissues, expression of this group of genes can be induced in somatic contexts in response to environmental or toxicological insults that perturb DNA methylation. Widespread changes in DNA methylation have been reported to occur in numerous cancers [221, 222], and genetic instability caused by retrotransposon activity can drive tumourigenesis [19]. The potential functional roles of the germline genome defence genes in hypomethylated somatic contexts is clearly an exciting area that requires further investigation.

## Consequences of failures in genome defence

Defects in germline genome defence might be expected to result in increased rates of retrotransposition, and new retrotransposon integrations being passed on to the next generation. However, mutations in most of the germline genome defence genes result in male infertility, making it difficult to assess de novo retrotransposition rates in their offspring. Curiously, many of the germline genome defence mutants have strong phenotypic similarities and common arrest points in spermatogenesis (Fig. 4). De-repression of retrotransposons might therefore have additional and more immediate consequences for germ cell development than generating new retrotransposition events in the next generation.

### Consequences of mutations in the DNA methylation machinery

At a cellular level, *Dnmt3l* ^−*/*−^ mice display defective spermatogonial proliferation, delayed entry into meiosis and arrested meiotic progression at pachytene in young adults. Older mutant adults become completely azoospermic due to progressive loss of spermatogonia, suggesting defects in the maintenance of spermatogonial stem cells [163, 223]. Although *Dnmt3l* ^−*/*−^ mutant prospermatogonia have defects in DNA methylation of multiple genomic features [152, 163], it is not clear how DNA hypomethylation causes the spermatogonial stem cell or meiotic phenotypes in these mice.

Impaired maintenance of spermatogonial stem cells and delayed entry into meiosis also occur in *Dnmt3a* ^−*/*−^ germ cells, whereas *Dnmt3b* ^−*/*−^ germ cells have no severe disruptions in spermatogenesis and are able to form functional sperm [159, 160]. Thus, methylation at DNMT3A-specific targets such as B1 SINE retrotransposons or some imprinted genes might be important for spermatogonial stem cell maintenance and timing of meiotic entry, but hypomethylation at DNMT3B-specific targets such as satellite sequences [152] does not disrupt spermatogenesis. Notably, the meiotic arrest seen in *Dnmt3l* ^−*/*−^ testes is not recapitulated in either the *Dnmt3a* ^−*/*−^ or *Dnmt3b* ^−*/*−^ single-knockout male germ cells [159, 160]. Thus, hypomethylation at redundant genomic targets of DNMT3A and DNMT3B, such as LINE-1 retrotransposons, IAP retrotransposons or some imprinted genes [152], are likely to be responsible for the meiotic defects in *Dnmt3l* ^−*/*−^ testes.

The meiotic arrest evident in *Dnmt3l* ^−*/*−^ spermatocytes is characterised by defective synaptonemal complex formation, with widespread chromosome asynapsis and non-homologous synapsis [163]. Recombination proteins RAD51 and RPA1 localise to chromosome axis-associated foci at a similar frequency in wild-type and *Dnmt3l* ^−*/*−^ spermatocytes [224], indicating that the chromosome synapsis defect is not a downstream consequence of a failure to initiate meiotic recombination. The presence of asynapsed chromosomes in *Dnmt3l* ^−*/*−^ pachytene spermatocytes sequesters the transcriptional silencing machinery, impairing meiotic sex chromosome inactivation and leading to spermatocyte apoptosis [224]. The meiotic arrest in *Dnmt3l* ^−*/*−^ testes is perhaps surprising given that expression of *Dnmt3l* is so low during meiosis [223], and presumably reflects defects in de novo methylation that arise earlier in the foetal prospermatogonia.

Although *Dnmt3l* ^−*/*−^ male mice have numerous cellular defects in spermatogenesis, meiosis and oogenesis proceed normally in females, notwithstanding the failure in maternal imprinting [164, 165]. The differential requirement for DNMT3L in meiotic chromosome synapsis between males and females likely reflects de novo methylation occurring before the initiation of meiosis in males, but after meiotic chromosome synapsis is complete in females [152, 153]. Oocytes therefore have low levels of genomic DNA methylation while they progress through the early stages of meiotic prophase [39]. The low level of DNA methylation that is present in meiotic oocytes appears to be required for normal chromosome synapsis and progression through meiosis. *Lsh* ^−*/*−^ oocytes have reduced levels of DNA methylation, and, although these oocytes initiate meiotic recombination normally, they fail to synapse their homologous chromosomes properly [172]. The meiotic defects in hypomethylated *Lsh* ^−*/*−^ oocytes bear some similarity to those in hypomethylated *Dnmt3l* ^−*/*−^ spermatocytes [163, 172, 224]. Thus, even though there are sex-specific differences in the mechanisms that generate the correct DNA methylation patterns for meiosis, reducing DNA methylation has similar consequences for meiotic chromosome synapsis in both spermatocytes and oocytes.

### Consequences of mutations in the PIWI–piRNA system

The PIWI–piRNA pathway acts genetically upstream of *Dnmt3l*-dependent de novo DNA methylation, and therefore many of the genome defence genes that are important for PIWI–piRNA function in foetal prospermatogonia share cellular spermatogenic defects with *Dnmt3l* ^−/−^ mice. Like *Dnmt3l* ^−*/*−^ testes, self-renewal/maintenance of spermatogonial stem populations are reduced in *Mili* ^−*/*−^ testes and in *Miwi2* ^−*/*−^ testes [177, 198]. The male germ cells that progress to meiosis in *Miwi2* ^−*/*−^ testes are able to initiate meiotic recombination, but chromosome synapsis is severely defective in these cells [177]. Interestingly, these meiotic defects arise some time after *Miwi2* is expressed during spermatogenesis (Fig. 4), suggesting that defects in *Miwi2* ^−*/*−^ prospermatogonia are being heritably transmitted through multiple rounds of mitosis. *Mili* ^−*/*−^ spermatocytes also fail to progress through pachytene, although the precise nature of the meiotic defects in these cells has not been determined [196]. At least some *Mili* ^−*/*−^ single-knockout and *Mili* ^−*/*−^ *Miwi* ^−*/*−^ double-knockout spermatocytes appear to successfully synapse their chromosomes, and progress further into pachytene than *Miwi2* ^−*/*−^ spermatocytes [177, 196, 225]. As MILI is required for localisation of MIWI2 to cytoplasmic granules and accumulation of the MIWI2-interacting piRNA population [176], it is not clear whether the apparent differences between the *Mili* ^−*/*−^ and *Miwi2* ^−*/*−^ meiotic phenotypes reflect complex interactions between these PIWI proteins during meiosis, or differences in the phenotypic analyses. Taken together, these data suggest that the PIWI–piRNA pathway and de novo DNA methylation in foetal prospermatogonia facilitate chromosome synapsis and progression through pachytene in postnatal meiotic spermatocytes.

Male mice with mutations in other genes required for piRNA biogenesis or PIWI–piRNA function, including *Mov10l1*, *Asz1*, *Mvh* and *Mitopld*, all have defects in progression through the zygotene/pachytene stages of meiotic prophase where chromosome synapsis occurs [190, 192, 193, 212, 226]. Where studied, chromosome synapsis is defective in these mutant spermatocytes, and the meiotic phenotype resembles *Miwi2* ^−*/*−^ mice [192, 193]. Female mice are fertile for each of these mutants, suggesting that chromosome synapsis is able to occur in meiotic oocytes in the absence of PIWI–piRNA function. *Mael* is also implicated in PIWI–piRNA pathways for germline genome defence, and is one of the few germline genome defence mutants where there is evidence of novel retrotransposition events taking place in the germline [191]. In the absence of MAEL, male mice are infertile, have defects in meiotic chromosome synapsis, and arrest at pachytene. A large increase in the levels of DNA damage is present in *Mael* ^−*/*−^ spermatocytes, and elegant genetic experiments have shown that the generation of DNA damage in *Mael* ^−*/*−^ spermatocytes is independent of the SPO11 endonuclease that generates the meiotic DNA double-strand breaks required for recombination [191]. The presence of nuclear LINE-1 ORF1 protein correlates with high levels of the DNA damage marker γH2AX in *Mael* ^−*/*−^ spermatocytes, implying that novel DNA damage is being generated by retrotransposons integrating into the genome.

Maintaining suppression of retrotransposons during spermatogenesis appears to be important even after meiotic chromosome synapsis. Spermatogenesis in *Miwi* ^−*/*−^ testes arrests at the round spermatid stage with increased abundance of LINE-1 transcripts, and elevated DNA damage [195, 199]. Impairing post-pachytene piRNA populations by conditionally deleting *Mov10l1* in pachytene spermatocytes also causes elevated DNA damage in round spermatids and spermatogenic arrest at this stage [201]. Although increased DNA damage and round spermatid arrest appear to be a consequence of a loss of piRNA-dependent MIWI slicer activity [199], further work is needed to determine whether these cellular phenotypes are caused by increased retrotransposon activity, or by other changes in these mutants. In contrast to spermatogenesis, mutations in the PIWI–piRNA system do not have severe defects on post-pachytene progression through oogenesis, despite *Mili* ^−*/*−^ oocytes exhibiting a modest increase in retrotransposon expression [197].

### Consequences of mutations in other germline genome defence mechanisms

DAZL is a germ cell-specific RNA binding protein that is essential for fertility in both male and female mice [217, 227–229]. Spermatogonial differentiation is greatly impaired in *Dazl* ^−*/*−^ testis, and, although a small proportion of germ cells progress to meiosis, they arrest at pachytene [230]. Various genes have been identified that are translationally regulated by DAZL, including the germline genome defence genes *Mvh* and *Tex19.1*, and genes known to be important for meiosis such as *Sycp3* [227, 228]. As DAZL translationally regulates *Mvh* and *Tex19.1*, it is likely that impaired translation of these genome defence genes accounts for some aspects of the *Dazl* ^−*/*−^ phenotype.

Unlike many of the other germline genome defence mutants, loss of *Tex19.1* causes defects during both oogenesis and spermatogenesis [213]. Approximately half the *Tex19.1* ^−*/*−^ pachytene spermatocytes have chromosome synapsis defects and exhibit apoptosis at this stage, while two-thirds of the nuclei progressing through to metaphase I contain at least one set of unpaired univalent chromosomes. Although the retrotransposon de-repression, chromosome asynapsis and pachytene arrest in *Tex19.1* ^−*/*−^ males are all shared with the other male germline genome defence mutants, the spectrum of retrotransposons de-repressed in *Tex19.1* ^−*/*−^ testes is distinct from PIWI-piRNA pathway mutants [213]. TEX19.1 is stabilised by the E3 ubiquitin ligase UBR2 [231], but no PIWI–piRNA pathway components have been connected to TEX19.1 or UBR2. Interestingly, the de-repression of MMERVK10C retrotransposons in *Tex19.1* ^−*/*−^ testes occurs in pachytene spermatocytes [213]; however, it is currently unclear what causes the chromosome synapsis defects in *Tex19.1* ^−*/*−^ spermatocytes.

### Mechanisms linking retrotransposon de-repression to meiotic chromosome asynapsis

Mutations in germline genome defence genes tend to cause male-specific defects in chromosome synapsis and arrest meiosis during pachytene (Fig. 4), although most female mutants do not have a severe phenotype at this stage of meiosis. The cause of the chromosome asynapsis in these male mutants is not well understood. Where reported, DNA double-strand breaks are generated at the start of meiosis in the genome defence mutants, indicating successful initiation of meiotic recombination [177, 191, 213, 224]. These meiotic DNA double-strand breaks repair as chromosomes synapse, but remain unrepaired on asynapsed regions [191, 213, 224], as would be expected from analysis of other meiotic mutants [232]. One informative feature of the chromosome asynapsis in the germline genome defence mutants is that the asynapsed chromosomes in these mutants are not paired, suggesting that the asynapsis is caused by a defect in the homology search rather than synaptonemal complex assembly.

Interestingly, *Mael* ^−*/*−^ mutants have increased amounts of SPO11-independent DNA damage, possibly caused by increased retrotransposition of LINE-1 elements, and these SPO11-independent DNA breaks recruit the homologous recombination protein RAD51 [191]. Thus, the increased DNA damage could be sequestering RAD51 and other meiotic recombination proteins away from meiotic double-strand DNA breaks generated by SPO11, thereby impairing the homology search. While moderate levels of additional DNA double-strand breaks generated by ionising radiation are well tolerated in spermatocytes [233], there appears to be a level at which DNA double-strand breaks become disruptive to meiotic progression. *Atm* ^−*/*−^ spermatocytes exhibit a tenfold increase in DNA double-strand breaks, which is accompanied by chromosome asynapsis [234]. Reducing the number of DNA double-strand breaks in *Atm* ^−*/*−^ spermatocytes by reducing the dosage of *Spo11* rescues the asynapsis [234]. Therefore, increased retrotransposition creating elevated levels DNA double-strand breaks could be contributing to meiotic chromosome asynapsis in genome defence mutants.

Although *Mael* ^−*/*−^ spermatocytes have elevated levels of DNA double-strand breaks, not all genome defence mutants exhibit this phenotype. Unlike *Mael* ^−*/*−^ spermatocytes, γH2AX and RAD51 staining in *Dnmt3l* ^−*/*−^ spermatocytes is relatively normal [224]. An alternative explanation for the defects in the meiotic homology search in genome defence mutants is that global changes in chromosome structure or organisation caused by widespread DNA hypomethylation could be generating an inappropriate chromosomal environment for the homology search. Alternatively, it is possible that raised levels of retrotransposon proteins present in genome defence mutants might be sufficient to disrupt the cellular environment of the developing male germ cells. Proteins encoded by human LTR retrotransposons physically interact with endogenous transcription factors present in germ cells and can disrupt spermatogenesis when ectopically expressed in transgenic mice [235–237]. Physical interactions between retrotransposon proteins and germ cell proteins involved in the meiotic homology search could explain the meiotic chromosome asynapsis in germline genome defence mutants. Further work is clearly needed to dissect out the mechanism causing the spermatocyte asynapsis and male infertility in the genome defence mutants.

## Concluding remarks

Germline genome defence mechanisms would be expected to play a role in maintaining genome stability over evolutionary time scales. However, it is becoming apparent that mutations in germline genome defence genes cause infertility and defects in progression through meiosis that are precluding any analysis of genome stability in their offspring. The mechanistic explanation for this unexpected requirement for the germline genome defence genes is not understood, and it is not clear if the meiotic defects in these mutants are related to retrotransposon de-repression, or to uncharacterised meiotic functions of the germline genome defence genes. Imbalances between genome defence and retrotransposon activities could be causing infertility in mouse models in a similar way that incompatibilities between retrotransposon activity and germline genome defence systems cause sterility and hybrid dysgenesis in fruit flies [238]. It will be of interest to determine whether failures in germline genome defence systems, or their epigenetic regulation [239], could be contributing to infertility in humans.

The regulatory coupling of genome defence mechanisms to the potential for retrotransposon activity is an interesting paradigm that raises a number of questions. The epigenetic disruption and recovery screen used to identify these genes was performed in fibroblasts and used stringent thresholds for activation of gene expression [43]. Additional genome defence genes, such as *Mael* and *Mvh*, could be regulated by DNA methylation in germ cells but not identified in this screen due to the absence of tissue-specific transcription factors, or the presence of additional layers of regulation in fibroblasts. Therefore, more work is needed to better understand tissue-specific responses to epigenetic disruption. Furthermore, there are likely to be some differences between the genome defence mechanisms operating in different species. Indeed, human retrotransposons are de-repressed in transchromosomic mice, suggesting that mouse genome defence mechanisms cannot effectively suppress human retrotransposon sequences [240]. Therefore, although the links between DNA methylation, retrotransposon suppression and genome defence genes are starting to be uncovered in mice, further work will be required to investigate whether these associations exist in other species, and whether similar associations exist in general between different retrotransposon transcriptional silencing mechanisms and different groups of genome defence genes.

## Abbreviations

5hmC: 5-Hydroxymethyl cytosine
5mC: 5-Methyl cytosine
CGI: CpG island
ERV: Endogenous retrovirus
ES cells: Embryonic stem cells
H3K27Ac: Histone 3 lysine 27 acetylation
H3K27Me3: Histone 3 lysine 27 trimethylation
H3K4Me: Histone 3 lysine 4 methylation
H3K9Me3: Histone 3 lysine 9 trimethylation
H4K20Me3: Histone 4 lysine 20 trimethylation
IAP: Intracisternal A particle
LINE: Long interspersed nuclear element
LTR: Long terminal repeat
piRNA: PIWI-interacting RNA
SINE: Short interspersed nuclear element
siRNA: Small interfering RNA
